# Revenues and Profits From Medicare Patients in Hospitals Participating in the 340B Drug Discount Program, 2013-2016

**DOI:** 10.1001/jamanetworkopen.2019.14141

**Published:** 2019-10-30

**Authors:** Rena M. Conti, Sayeh S. Nikpay, Melinda B. Buntin

**Affiliations:** 1 Boston University Questrom School of Business, Boston, Massachusetts; 2 Department of Health Policy, Vanderbilt University School of Medicine, Nashville, Tennessee

## Abstract

**Importance:**

The federal 340B program lowers the acquisition cost of prescription drugs and places no limits on what hospitals charge payers. Congress established the program to allow 340B profits (the difference between payments and acquisition costs) to subsidize other safety-net services. Little is known about the magnitude of revenues and profits from the 340B program among participating hospitals.

**Objective:**

To report revenues and estimated profits from the 340B program that hospitals collect from Medicare and Medicare beneficiaries for outpatient clinic administration of prescription drugs covered under Medicare Part B.

**Design, Setting, and Participants:**

This cross-sectional descriptive study used 100% Medicare outpatient Part B claims from January 1, 2013, to December 31, 2016, from fee-for-service Medicare beneficiaries administered separately payable drugs at general acute care nonprofit or public hospitals without special payment designations. Claims data (N = 11 298 860) were aggregated to the hospital-year level (N = 6000) and linked to hospital finances and 340B participation from Medicare cost reports and the 340B covered entity list.

**Main Outcomes and Measures:**

Outcomes studied were revenue and estimated profits, assuming a 50% discount from 340B-discounted drug administrations to Medicare patients, as well as Medicare 340B profits relative to hospital net operating revenue, uncompensated care, and disproportionate share hospital payments.

**Results:**

During the study period, hospitals received approximately $2.1 billion in 340B revenue from Medicare in 2013, increasing to $3.7 billion in 2016. Estimated 340B profits from Medicare in 2016 totaled $1.9 billion, and per-hospital estimated 340B profits were $2.5 million but exhibited variability (median, $0.8 million; interquartile range, $0.1 million-$2.8 million). In 2016, median estimated 340B profits from Medicare were 0.3% (interquartile range, 0.1%-0.7%; mean, 0.4%) of hospital operating budgets and 9.4% (interquartile range, 1.8%-26.5%; mean, 16.6%) of hospital uncompensated care costs.

**Conclusions and Relevance:**

Estimated profits that hospitals derived from administering 340B-discounted drugs to Medicare patients are small compared with operating budgets yet substantial compared with uncompensated care costs for many hospitals. Revenue and profit estimates from 340B-discounted drugs represent a lower bound because data on revenue from the sale of outpatient retail dispensed drugs by hospital contract pharmacies and commercial insurer claims are not available.

## Key Points

**Question:**

How much revenue and estimated profit do hospitals participating in the 340B drug discount program collect from Medicare and Medicare beneficiaries for the outpatient clinic administration of prescription drugs covered under Part B?

**Findings:**

This cross-sectional study found that, in 2016, hospitals received $3.7 billion for the administration of drugs in the 340B discount program; assuming a 50% discount, profits totaled $1.9 billion. Median 340B profits from Medicare were 0.3% of hospital operating budgets and 9.4% of hospital uncompensated care costs.

**Meaning:**

Revenues and estimated profits from administering drugs in the 340B discount program to Medicare beneficiaries are small in terms of overall hospital budgets but substantial compared with uncompensated care costs.

## Teaser

This cross-sectional study reports revenues and estimated profits from the 340B drug discount program that hospitals collect from Medicare and Medicare beneficiaries for outpatient clinic administration of prescription drugs covered under Medicare Part B.

## Introduction

“Safety-net” providers—hospitals and outpatient clinics serving large uninsured and low-income populations—are subsidized to sustain or increase the supply of medical care to patients with limited ability to pay. In 1992, Congress created the 340B drug discount program to support safety-net hospitals. The 340B drug discount program provides acquisition cost discounts for outpatient drugs and places no limits on what participating hospitals charge payers for discounted drugs.^1^ According to a report from the Office of the Inspector General, most 340B discounts range from 25% to 75% of the amount Medicare pays for physician-administered drugs and apply to all drugs purchased by the safety-net provider, not just drugs for patients who are uninsured or low income.^2,3^ Congress’s intent in establishing the 340B program was to reduce hospitals’ costs of largely uncompensated drug-based care and allow hospitals to subsidize other safety-net activities with profits generated from outpatient drugs.

General acute care nonprofit and public hospital participation in the 340B program has increased rapidly in the past decade, as have prices for oncology drugs and other drugs commonly administered by physicians in the outpatient setting.^2^ Growth has led federal and state policy makers and other stakeholders to argue for greater program oversight.^4^ Current program rules do not require hospitals to report the amount of 340B revenues or profits they generate from payers. The Centers for Medicare & Medicaid Services recently proposed lowering Medicare reimbursements for 340B-eligible drugs covered under Part B to reflect discounts, in part to save beneficiaries money given the benefits’ deductible, 20% cost sharing requirement, and lack of a catastrophic limit.^5,6,7,8^ Evaluating the potential intended and unintended consequences of current and future reforms^9,10,11,12,13^ to oversight and implementation of the 340B program requires empirical evidence of the amount of revenues and profits the 340B program derives from Medicare and Medicare beneficiaries.

We report Medicare Part B–related revenues and estimated profits from 340B-discounted drugs among hospitals participating in the program between 2013 and 2016. We compare these amounts with hospital self-reported finance statistics and other government subsidies provided to sustain hospitals’ provision of safety-net services.

## Methods

The analytic sample included 1500 hospitals observed from January 1, 2013, to December 31, 2016 (N = 6000 [1500 hospitals observed in each year of the study period aggregated]), and 11 298 860 physician-administered, Part B–covered drug claims reimbursed by Medicare and Medicare beneficiaries. The study was reviewed and given exempt status by the Vanderbilt University Institutional Review Board because the data were not gathered on human participants. This study followed the Strengthening the Reporting of Observational Studies in Epidemiology (STROBE) reporting guideline.

The analytic sample was constructed in the following way (eTable 1 in the Supplement): we started with 100% Medicare outpatient claims for fee-for-service Medicare beneficiaries, which capture all drug administrations covered and reimbursed by Medicare’s Part B outpatient benefit. We identified outpatient drug administrations using J-codes observed in the revenue center file of each claim and extracted the amount the hospital billed the Medicare program and the Medicare beneficiary for the drug administration, the date of the administration, and the Centers for Medicare & Medicaid Services’ hospital identifier.

We excluded drug administrations by non–general acute care hospitals such as critical access hospitals, administrations by hospitals in the US territories, and administrations that were paid by both Medicare and Medicaid or paid by Medicare and another primary (non-Medigap) plan. We then aggregated included claims to the hospital-year level. We created separate measures of total drug administrations and Medicare revenues for anticancer drugs identified using a previously published J-code list.^14^

We further restricted our hospital-year-level data set to exclude hospitals only observed in fewer than all 4 years, for-profit hospitals, hospitals with missing or negative payment information, hospitals that quit the 340B program before the end of our study, and freestanding hospitals or hospitals with special Medicare payment designations.^15^ These exclusions included claims from freestanding cancer hospitals, rural referral centers, Medicare-dependent hospitals, sole community hospitals, and hospitals with more than 1 of these special designations.

We linked sample hospitals to the Health Resources and Services Administration 340B covered entity list to identify hospital participation and dates of participation in the 340B program. We also linked the data to hospital finance and other characteristics reported in Medicare cost reports. These annual reports are completed by all hospitals that receive reimbursement from Medicare. We included net hospital operating revenues, which is the amount hospitals receive in revenue from patient care, net of contractual allowances, or insurer discounts^16^; hospital operating margins, which were equal to net operating revenue, less operating expenses, divided by net operating revenue; and uncompensated care, which is defined as the sum of hospital charity care costs and bad debt costs.^17^ All 3 of these variables are measured with error, and there are often outlier values.^18^ Consequently, we used winsorization to top-code values in the top 10th percentile of each measure, following standard practice.^19^

Hospitals receive additional federal and state subsidies to provide safety-net services, commonly referred to as disproportionate share payments. The Medicare cost reports are the official source of data for Medicare (federal) disproportionate share hospital (DSH) payments; however, they do not contain information on Medicaid (state) DSH payments. Consequently, we used 2013 state-level Medicaid DSH audit reports to obtain information on how much each hospital receives in state DSH payments. State DSH payments were not available past 2014, so we estimated Medicaid DSH payments in subsequent years based on 2013 data using hospital characteristics.

We report revenues that hospitals received for outpatient drug administrations eligible for 340B discounts and paid by Medicare and Medicare beneficiaries. Revenues include both amounts paid directly to hospitals by Medicare and amounts expected from patients or their Medigap insurers and does not include reimbursements for the administration itself, related facility fees, and physician time.^20^ We estimated profits derived from these revenues by assuming the discounted 340B program price is 50% of Medicare reimbursement, based on prior research by the Office of the Inspector General,^3^ because we did not have access to statutorily based acquisition cost estimates for each drug reimbursed under Part B. We report estimated profits and also estimated profits relative to key hospital financial indicators for safety-net hospitals: net operating revenues, uncompensated care, and combined federal and state DSH payments.

One important limitation of our study is that we did not have access to claims for drugs dispensed by outpatient pharmacies that were eligible for 340B discounts by participating hospitals and their contract pharmacy relationships.^9^ Hospitals participating in the 340B program may generate profits from dispensing discounted drugs to Medicare beneficiaries through these pharmacies as well. To understand how much this limitation may influence our results, we used the Health Resources and Services Administration’s 340B pharmacy list to identify the number of contract pharmacies each sample hospital had in each year.

### Statistical Analysis

Sample construction and analyses were conducted using Stata, version 14 (StataCorp). Totals, means, medians, and interquartile ranges for outcomes as defined above were calculated and reported. No statistical adjustments, other than winsorization, were made.

## Results

Hospital participation in the 340B program increased between 2013 and 2016 from 614 to 748 (Table).^17,21,22^ During this period, the number of outpatient drug administrations also increased, particularly among oncology drugs. There are several notable differences across 340B and non-340B hospitals in 2013, the baseline year of our study. Hospitals participating in the 340B program administered many more outpatient drugs under Medicare for all drugs and for oncology drugs specifically. Participants in the 340B program were larger in terms of both beds and net operating revenue and had lower operating margins and higher amounts of uncompensated care. Participants in the 340B program also received more DSH payments than did nonparticipating hospitals. Over time, 340B participants’ net operating income increased and became more financially stable, with higher operating margins and lower uncompensated care. Over time, both Medicare and imputed Medicaid DSH payments decreased slightly among participants.

**Table.** Characteristics of 340B Participating Hospitals in 2013 and 2016 Compared With Nonparticipating Hospitals in 2013^a^

Characteristic	340B Participating Hospitals				Nonparticipating Hospitals	
	2013 (n = 614)		2016 (n = 748)		2013 (n = 886)	
	Mean	Median (IQR)	Mean	Median (IQR)	Mean	Median (IQR)
Medicare outpatient drug administrations, No.	2497	973 (303 to 3144)	2799	883 (220 to 3084)	1452	597 (208 to 1588)
Medicare outpatient oncology drug administrations, No.^b^	849	180 (17 to 1034)	996	242 (18 to 1251)	428	33 (0 to 275)
General acute care beds, No.^c^	279	236 (138 to 375)	312	216 (122 to 351)	183	146 (80 to 236)
Net operating revenue, millions, $^d^	446	326 (144 to 580)	483	334 (150 to 637)	269	197 (105 to 326)
Operating margin, %^e^	−9	−3 (−11 to 3)	−7	−2 (−11 to 5)	−2	0 (−6 to 6)
Uncompensated care costs, millions, $^f^	29	14 (6 to 31)	24	10 (4 to 24)	9	7 (3 to 12)
DSH, millions, $^g^						
Medicaid	9	0 (0 to 4)	8	5 (1 to 11)	1	0 (0 to 0)
Medicare	10	8 (3 to 13)	7	5 (2 to 11)	2	1 (0 to 3)

Abbreviations: DSH, disproportionate share hospital; IQR, interquartile range.

^a^ Calculations based on data constructed and described in eTable 1 in the Supplement.

^b^ Outpatient drug administrations are the total number of drug administrations across all J-codes, and outpatient oncology drug administrations are drug administrations for oncology J-codes as identified by a previously published report.^21^

^c^ Beds are the number of general and pediatric inpatient beds.

^d^ Net operating revenue is revenue from patient operations net of contractual allowances, which are negotiated discounts from insurers.

^e^ The operating margin is net operating revenue, less expenses, divided by net operating revenue, and it represents the share of net operating revenue that can be retained as profits.

^f^ Uncompensated care is defined as the cost of bad debt and charity care. The definition of uncompensated care changed for hospital cost report periods beginning after October 2016. Therefore, some fraction of these data may reflect the new definition.^17^

^g^ Medicare DSH payments are the sum of empirically justified DSH and uncompensated care DSH payments.^22^ Medicaid DSH payments come from Medicaid DSH audit reports and were not available for 2016; 2013 data were used to estimate 2016 Medicaid DSH payments.

**Figure 1.**
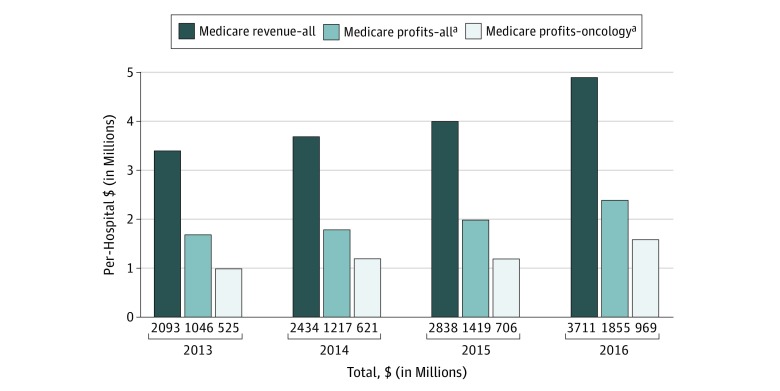
Hospital Revenues and Estimated Profits From the Outpatient Administration of Medicare Part B–Covered, Physician-Administered Drugs Paid by Medicare and Medicare Beneficiaries by Total per Hospital and Restricted to Oncology Only, 2013-2016 Per-hospital revenue and estimated profits are shown, and total revenue and estimated profits in millions of dollars are shown under the bars. Calculations are based on data constructed and described in eTable 1 in the Supplement. The sample includes nonprofit and public general acute care hospitals participating in the 340B program in each year. Revenue and estimated profits are defined as the amount hospitals receive for outpatient physician-administered drug treatment covered under Part B and paid by Medicare and Medicare beneficiaries. ^a^The figures presented are estimated based on an assumption that the cost of drugs under the 340B program are equal to 50% of revenue. Outpatient oncology drug administrations are drug administrations for oncology J-codes as identified by a previously published report.^21^

In 2013, hospitals participating in the 340B program received approximately $2.1 billion in revenue from Medicare for 340B-eligible drugs (Figure 1^21^; eTable 2 in the Supplement). On a per-hospital basis, the median revenue per hospital was $1.2 million (interquartile range [IQR], $0.2 million-$3.9 million; mean, $3.4 million). By 2016, revenue from 340B-eligible drug administrations increased to $3.7 billion across all hospitals and a median of $1.5 million per hospital (IQR, $0.3 million-$5.6 million; mean, $5.0 million).

Estimated profits among all hospitals from 340B-discounted drugs also increased from approximately $1.1 billion in 2013 to $1.9 billion in 2016. Median profits per hospital increased from $0.6 million in 2013 (IQR, $0.1 million-$1.9 million; mean, $1.7 million) to $0.8 million in 2016 (IQR, $0.1 million-$2.8 million; mean, $2.5 million). Total hospital estimated profits from administrations of anticancer drugs increased from $525.5 million in 2013 to $969.8 million in 2016 and on a median per-hospital basis from $0.4 million (IQR, $0.1 million-$1.4 million; mean, $1.1 million) in 2013 to $0.6 million (IQR, $0.1 million-$2.2 million; mean, $1.7 million) in 2016. In total and on a per-hospital basis, estimated profits from oncology drugs were higher and increased more quickly than profits for all drugs between 2013 and 2016.

Next, we considered the size of estimated profits from administering 340B-eligible drugs to Medicare patients relative to net hospital operating revenues (Figure 1; eTable 2 in the Supplement). Relative to net operating revenues, estimated 340B profits are small. For the median hospital, profits as a share of net operating revenue represented less than 1%. In 2013, profits as a share of net operating revenue were 0.2% of net operating revenues (IQR, 0.1%-0.5%; mean, 0.3%), increasing to 0.3% (IQR, 0.1%-0.7%; mean, 0.4%) by 2016. Even among hospitals at the 75th percentile, profits were only 0.7% of net operating revenues by 2016.

**Figure 2.**
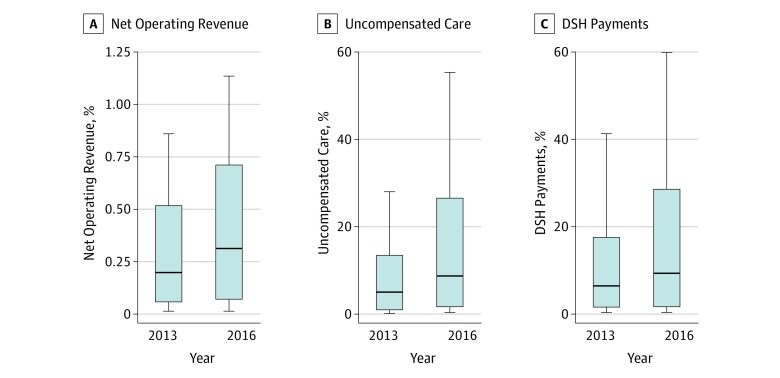
Size of Estimated Profits From the Outpatient Administration of Medicare Part B–Covered, Physician-Administered Drugs Paid by Medicare and Medicare Beneficiaries Relative to Benchmarks in 2013 and 2016 Calculations based on data constructed and described in eTable 1 in the Supplement. The sample includes nonprofit and public general acute care hospitals participating in the 340B program in each year. One hundred percent of Medicare outpatient Part B claims are linked to the 340B hospital covered entity list, Medicare hospital cost reports, and 2013 Medicaid disproportionate share hospital (DSH) audits. The top and bottom borders of each box represent the 75th and 25th percentile, respectively, and the center line inside each box represents the median. The whiskers represent 1.5 times the interquartile range. A, Estimated profits from administering drugs to Medicare patients, divided by net operating revenue. Net operating revenue is revenue from operations less insurer discounts. B, Estimated profits as a share of uncompensated care costs. Uncompensated care is defined as the sum of charity care costs and bad debt costs. C, Estimated profits as a share of hospital disproportionate share payments in Medicare and Medicaid. Medicaid DSH payment data were not available and so 2016 amounts are estimated using 2013 data. Profits are estimated based on an assumption that the cost of drugs under the 340B program is equal to 50% of revenue.

Although profits from administering 340B-eligible drugs to Medicare patients are small relative to the overall operating budget, they are sizeable when measured relative to other safety-net service benchmarks (Figure 2; eTable 2 in the Supplement). Median 340B profits per hospital were 6.3% of uncompensated care costs in 2014 (IQR, 1.3%-19.2%; mean, 13.1%) and 9.4% of uncompensated care costs in 2016 (IQR, 1.8%-26.5%; mean, 16.6%). This large increase reflects nationwide reductions in uncompensated care in 2014 after the implementation of the Patient Protection and Affordable Care Act’s coverage expansions.^23^ For hospitals at the 75th percentile, Medicare profits from 340B drug administrations were even larger, increasing from 19.2% in 2014 to 26.5% in 2016.

Estimated profits from administering 340B drugs to Medicare patients were similarly sized when measured relative to estimated DSH payments from Medicare and Medicaid. Median estimated Medicare profits per hospital were 6.4% of DSH payments in 2013 (IQR, 1.6%-17.6%; mean, 12.7%) and 9.4% of DSH payments in 2016 (IQR, 1.6%-28.5%; mean, 18.2%). Similar to uncompensated care, 340B profits from Medicare patients were larger at the 75th percentile in 2013 (17.6%) and even larger in 2016 (28.5%).

**Figure 3.**
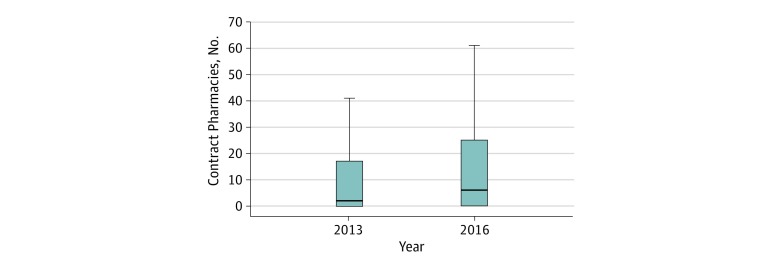
Distribution of Contract Pharmacy Relationships Among 340B Participating Hospitals, 2013-2016 Calculations based on data constructed and described in eTable 1 in the Supplement. The sample includes nonprofit and public general acute care hospitals participating in the 340B program in each year. Contract pharmacies were identified and counted using the 340B covered entity list, which reports the existence and name of each active contract pharmacy relationship that the 340B participating hospital has in each year. Box plots display the number of contract pharmacy relationships among 340B hospitals in 2013 and 2016. The top and bottom borders of each box represent the 75th and 25th percentile, respectively, and the center line inside each box represents the median. The whiskers represent 1.5 times the interquartile range.

In the absence of outpatient pharmacy data, we considered the number of pharmacies that participated in 340B hospital contracts between 2013 and 2016 (Figure 3; eTable 2 in the Supplement). The median 340B hospital in our sample had 2 contract pharmacies in 2013 (IQR, 0-17; mean, 21) and 6 by 2016 (IQR, 0-25; mean, 20.7). At the 75th percentile, the number of contracts was 17 in 2013, increasing to 25 per hospital by 2016. However, the 25th percentile hospital had no contract pharmacies during the study period.

In eTable 3 in the Supplement, we report 340B hospital participation, 340B revenues, estimated profits, and other finance characteristics by US states and the District of Columbia. Maryland, New Hampshire, North Dakota, and Wyoming had no participating 340B hospitals. Hospitals in 9 states (California, Florida, Georgia, Illinois, Michigan, North Carolina, New York, Pennsylvania, and Tennessee) accrued more than half of all 340B revenues and greater than $150 million each; however, hospitals in several other states (Kansas, Montana, Nebraska, Oklahoma, South Dakota, Vermont, and Virginia) had median 340B hospital revenues higher than $8 million each, far exceeding the national median. States with the highest total or median hospital 340B revenues and estimated profits varied considerably in other finance statistics and the number of contract pharmacy arrangements.

## Discussion

We found that acute care nonprofit and public hospitals and drugs covered by Medicare Part B are increasingly eligible for 340B discounts, and revenues and estimated profits from Medicare patients for hospitals participating in the 340B program are large and increasing over time. Our results suggest that the Office of the Inspector General’s focus on oncology drugs alone in a previous report underestimates 340B revenues and profits that hospitals make from Medicare Part B reimbursements. Other assessments of hospital behavior in expectation of 340B profit generation from oncology drug treatment paid by Medicare Part B^24,25^ might also provide a limited perspective. Moreover, our results suggest that there is substantial variability in the benefits of participation in the 340B program nationwide and by state. In the wider context of hospital finances, estimated 340B profits from Medicare Part B amounted to less than 1% of net hospital operating margins in all years and are smaller than other government subsidies to fund safety-net care, but profits appear to be increasing.

Results of our descriptive study have important implications for efforts to reform the 340B program. First, in the 2020 proposed budget released March 2019, the White House announced its interest in setting enforceable standards of program participation and requiring all covered entities to report on the amount of revenue and profits they generate from program participation and their use of program savings.^12^ This proposal is similar to a 2018 bill introduced into the US Senate sponsored by Senator Grassley, motivated by reforms in part related to the effect the program was having on Medicare patient drug spending.^13^ California already imposes additional reporting requirements on hospitals,^29^ and Ohio and Wisconsin, among other states, have recently proposed implementing increased oversight for hospitals participating in the 340B program, including additional reporting of 340B revenues and profits. Our efforts, in combination with these existing efforts, suggest that it is feasible for hospitals to report 340B revenue and estimated profits overall by payer and by therapeutic drug class and for regulators and other stakeholders to oversee the production of these reports. Given the caveats discussed herein, the data sources and assumptions used by the hospitals to report these statistics should be subject to standardization.

Second, in 2018, the US Department of Health and Human Services reduced Medicare payments by 22% for prescription drugs administered by a physician on an outpatient basis that were covered under Part B for acute care, not-for-profit hospitals participating in the 340B program.^10^ In late 2018, a federal court found that the US Department of Health and Human Services had exceeded its authority by making these cuts but did not rescind their application retrospectively or make any final determination regarding their future implementation.^11^ Congress has since delayed implementing the cuts. One potential benefit of Medicare payment cuts would be to reduce Medicare beneficiary payments for drug care administered by physicians to outpatients. Future work should quantify the effect of these reimbursement changes on Medicare beneficiary out-of-pocket costs associated with physician-administered, drug-based treatment for cancer and other conditions.

Finally, the hospitals with the highest Medicare revenues and net operating margins derived from the 340B program and the geographic areas with the highest 340B participation and highest 340B revenue among eligible hospitals are likely to be the ones most affected by 340B reforms. Although several 340B hospital trade associations have released voluntarily reported measures of 340B revenue benefits and likely effects of reform,^30^ future work should independently and systematically quantify the distributional effect of implementing reforms on hospital finances and operations. Data on revenues from sources beyond Medicare are needed to understand the association between estimated 340B profits and other hospital characteristics and to understand the causal effect of 340B eligibility on total hospital revenues, profits, operating margins, and measures of community benefit at the national and state level.

### Limitations and Strengths

These comparative statistics must be interpreted cautiously, as our results represent a lower bound on hospital revenues and profits from the 340B program for 2 reasons. First, we did not have access to claims for drugs dispensed by outpatient pharmacies that were eligible for 340B discounts by participating hospitals and their contract pharmacy arrangements.^9^ Our results suggest that revenues and profits from contract pharmacies might be an important source of funding for 340B hospitals, particularly in selected states. Second, we did not have access to drugs administered by physicians on an outpatient basis that are eligible for 340B discounts by hospitals reimbursed by commercial insurers and their beneficiaries. Based on 2016 National Health Expenditure Account data, commercial insurers comprise approximately 40% of all retail prescription drug expenditures^26^; thus, we expect that revenues and profits among hospitals using 340B-discounted drugs to care for commercially insured patients may be large.

Nevertheless, the positive attributes of our approach were that the data used are widely available to researchers, and our acquisition cost assumptions were justified using government reports. Because we are using actual reimbursement amounts paid and a reliable method for linking Part B claims to 340B participation, our methods also did not rely on the assumptions^27^ used by a recently published estimate of 340B size derived from drugs covered by Medicare Part D.^28^ A complete view of hospital 340B revenues and estimated profits cannot be achieved without additional data, much of which are not currently publicly available.

## Conclusions

This cross-sectional study found that the estimated profits that hospitals derived from administering 340B-discounted drugs to Medicare patients are small compared with operating budgets yet substantial compared with uncompensated care costs for many hospitals.

## Notes

Supplement. **eTable 1.** CONSORT Diagram of 340B Hospitals Included in the Analytic Sample **eTable 2.** Detailed Statistics Underlying Figures 1-3 **eTable 3.** State 340B Hospital Characteristics, 340B Revenues and Estimated 340B Profits Off the Outpatient Administration of Medicare Part B–Covered Physician-Administered Drugs Paid by Medicare and Medicare Beneficiaries in 2016
