# Supplementary material for: Revenues and Profits From Medicare Patients in Hospitals Participating in the 340B Drug Discount Program, 2013-2016
Source: JAMA Netw Open. 2019 Oct 30;2(10):e1914141. doi: 10.1001/jamanetworkopen.2019.14141 (PMC6824218; doi:10.1001/jamanetworkopen.2019.14141)
Supplement: Supplement. — eTable 1. CONSORT Diagram of 340B Hospitals Included in the Analytic Sample eTable 2. Detailed Statistics Underlying Figures 1-3 eTable 3. State 340B Hospital Characteristics, 340B Revenues and Estimated 340B Profits Off the Outpatient Administration of Medicare Part B–Covered Physician-Administered Drugs Paid by Medicare and Medicare Beneficiaries in 2016 [file jamanetwopen-2-e1914141-s001.pdf]

Supplementary Online Content

Conti RM, Nikpay SS, Buntin MB. Revenues and profits from Medicare patients in hospitals participating in the 340B drug discount program, 2013-2016. *JAMA Netw Open.* 2019;2(10):e1914141. doi:10.1001/jamanetworkopen.2019.14141

eTable 1. CONSORT Diagram of 340B Hospitals Included in the Analytic Sample
eTable 2. Detailed Statistics Underlying Figures 1-3
eTable 3. State 340B Hospital Characteristics, 340B Revenues and Estimated 340B Profits Off the Outpatient Administration of Medicare Part B–Covered Physician-Administered Drugs Paid by Medicare and Medicare Beneficiaries in 2016

This supplementary material has been provided by the authors to give readers additional information about their work.

© 2019 Conti RM et al. *JAMA Network Open.*

eTable 1. CONSORT Diagram of 340B Hospitals Included in the Analytic Sample

	Claims	Hospital-Years	Excluded
**Start Claims Data: All J-codes in 100percent Outpatient Claims**	**362534553**		
Exclude J codes that are not separately payable	20127903		-342406650
Exclude non-OPPS Payment	20127819		-84
Exclude non-acute care hospitals	20127819		0
Exclude hospitals in U.S. territories	19922556		-205263
Exclude claims that are not paid by Medicare	19920062		-2494
Exclude claims that are only paid by other primary insurance	19777944		-142118
Exclude those with no information on Medicaid dual status	19733932		-44012
**End Claims Data: Aggregate to Hospital-Year level data**		**12566**	
**Start Hospital Data:**		**12566**	
Exclude hospitals not observed in each year		11776	-790
Exclude for-profit hospitals		8848	-2928
Exclude hospitals with missing Medicare payment information		8844	-4
Exclude hospitals that quit 340B before 2016		8244	-600
Exclude freestanding cancer hospitals		8228	-16
Exclude rural referral centers		7656	-572
Exclude Medicare dependent hospitals		7292	-364
Exclude sole community hospitals		6468	-824
Exclude hospitals with multiple designations		6000	-468
**End: Hospital-year level data**	11298860	**6000**	

The figure describes the construction of the analytic dataset. We begin with 100 percent Medicare outpatient claims from 2013-2016 and select all J-codes, which indicate outpatient administered drugs. We exclude claims which are exempt from the outpatient prospective payment system, that are not separately payable, those that are not administered by physicians in non-acute care hospitals

© 2019 Conti RM et al. *JAMA Network Open.*

(such as critical access hospitals), and claims administered in the U.S. territories. We also exclude claims which Medicare declined to pay or claims for which there were two primary payers such as Medicaid and Medicare. Although this last step eliminated many patients with dual eligible status, we also limited our sample to individuals who were identified as non-dual eligibles in the Medicare beneficiary file. This exclusion is consistent with previously published estimates based on 340B eligible claims. We collapsed 19,733,932 claims that met our exclusion criteria to the hospital year level resulting in 12,566 hospital-year observations. We eliminated hospitals not observed in every year, hospitals that were for-profits (because they are ineligible for 340B), and hospitals that lost eligibility for 340B over our sample period. Finally, we eliminated hospitals that had at least one special Medicare payment designations or were free-standing cancer hospitals because they are ineligible to receive 340B discounts on drugs with any orphan indication. Our final sample included 1,500 unique hospitals and 6,000 hospital-year observations. The number of claims represented by this data was 11,298,860.

© 2019 Conti RM et al. *JAMA Network Open.*

eTable 2. Detailed Statistics Underlying Figures 1-3

Year	Variable	Mean	Median	25th Percentile	75th Percentile	Total
2013	Medicare Revenue ($ M)	3.4	1.2	0.2	3.9	2093.3
2013	Medicare Estimated Profits* ($ M)	1.7	0.6	0.1	1.9	1046.6
2013	Medicare Estimated Profits* - Oncology Drugs Only ($ M)	1.1	0.4	0.1	1.4	525.5
2013	Medicare Estimated Profits* Relative to Net Operating Revenue (%)	0.3	0.2	0.1	0.5	
2013	Medicare Estimated Profits* Relative to Uncompensated Care (%)	8.6	5.0	1.0	13.4	
2013	Medicare Estimated Profits* Relative to DSH Payments (%)	12.7	6.4	1.6	17.6	
2013	Contract Pharmacies	21.0	2.0	0.0	17.0	12900.0
2014	Medicare Revenue ($ M)	3.8	1.2	0.3	4.5	2434.2
2014	Medicare Estimated Profits* ($ M)	1.9	0.6	0.1	2.2	1217.1
2014	Medicare Estimated Profits* - Oncology Drugs Only ($ M)	1.2	0.4	0.1	1.6	621.6
2014	Medicare Estimated Profits* Relative to Net Operating Revenue (%)	0.3	0.2	0.1	0.6	
2014	Medicare Estimated Profits* Relative to Uncompensated Care (%)	13.1	6.3	1.3	19.2	
2014	Medicare Estimated Profits* Relative to DSH Payments (%)	11.8	5.5	1.1	18.6	
2014	Contract Pharmacies	22.5	6.0	0.0	22.0	14500.0
2015	Medicare Revenue ($ M)	4.1	1.3	0.3	4.6	2838.0
2015	Medicare Estimated Profits* ($ M)	2.0	0.7	0.1	2.3	1419.0
2015	Medicare Estimated Profits* - Oncology Drugs Only ($ M)	1.3	0.5	0.1	1.7	706.4
2015	Medicare Estimated Profits* Relative to Net Operating Revenue (%)	0.4	0.3	0.1	0.6	
2015	Medicare Estimated Profits* Relative to Uncompensated Care (%)	11.4	6.7	1.3	17.4	
2015	Medicare Estimated Profits* Relative to DSH Payments (%)	13.5	6.8	1.3	20.8	
2015	Contract Pharmacies	20.8	6.0	0.0	24.5	14400.0
2016	Medicare Revenue ($ M)	5.0	1.5	0.3	5.6	3711.9
2016	Medicare Estimated Profits* ($ M)	2.5	0.8	0.1	2.8	1856.0
2016	Medicare Estimated Profits* - Oncology Drugs Only ($ M)	1.7	0.6	0.1	2.2	969.8
2016	Medicare Estimated Profits* Relative to Net Operating Revenue (%)	0.4	0.3	0.1	0.7	
2016	Medicare Estimated Profits* Relative to Uncompensated Care (%)	16.6	8.6	1.8	26.5	
2016	Medicare Estimated Profits* Relative to DSH Payments (%)	18.2	9.4	1.6	28.5	
2016	Contract Pharmacies	20.7	6.0	0.0	25.0	15500.0

© 2019 Conti RM et al. *JAMA Network Open.*

Source: Authors calculations based on data constructed and described in eTable 1. The sample includes non-profit and public general acute care hospitals participating in 340B in each year. Contract pharmacies were identified and counted using the 340B provider list which reports the existence and name of each active contract pharmacy relationships the 340B participating hospital has in each year. Note: Revenue is defined as the amount hospitals receive for outpatient physician-administered drug treatment covered under Part B and paid by Medicare and Medicare beneficiaries. Profits are starred to remind readers that the figures presented are estimated based on an assumption that the cost of drugs under 340B are equal to 50 percent of revenue. Net operating revenue is revenue from patient operations net of contractual allowances, which are negotiated discounts from insurers. The operating margin is net operating revenue, less expenses, divided by net operating revenue, and it represents the share of net operating revenue that can be retained as profits. Uncompensated care is defined as the cost of bad debt and charity care. The definition of uncompensated care changed for hospital cost report periods beginning after October, 2016. Therefore some fraction of our data may reflect the new definition.[17] Medicare DSH payments are the sum of empirically justified DSH and uncompensated care DSH payments.[30] Medicaid DSH payments come from Medicaid DSH audit reports and were not available for 2016. We used 2013 data to predict 2016 Medicaid DSH payments.

eTable 3. State 340B Hospital Characteristics, 340B Revenues and Estimated 340B Profits Off the Outpatient Administration of Medicare Part B–Covered Physician-Administered Drugs Paid by Medicare and Medicare Beneficiaries in 2016

	No. hospitals participating	Medicare Revenue ($ M)					Medicare Estimated Profits* ($ M)					Net hospital operating revenue ($M)	Uncompensated care ($M)	DSH Payments	Medicare Estimated Profits* Relative to Net Operating Revenue	Medicare Estimated Profits* Relative to Uncompensated Care	Medicare Estimated Profits* Relative to DSH Payments	Contract Pharmacy Relationships			
State	(share eligible hospitals)	Total	Mean	Median	25th percentile	75th percentile	Total	Mean	Median	25th percentile	75th percentile	Total	Total	Total	Percent	Percent	Percent	Total	Median	25th percentile	75th percentile
AK	1 (100%)	2.1	2.1	2.1	2.1	2.1	1	1	1	1	1	671	17.1	9.5	0.2%	6.1%	11.0%	0	0	0	0
AL	12 (52%)	79.9	6.7	1.1	0.5	6.2	40	3	1	0	3	2659	155.6	144.2	1.5%	25.7%	27.7%	36	1	0	4
AR	4 (44%)	28.9	7.2	3.2	1.5	13.0	14	4	2	1	7	1897	47.1	65.6	0.8%	30.7%	22.0%	120	12	3	58
AZ	13 (56%)	56.1	4.3	2.7	0.1	4.4	28	2	1	0	2	4710	123.5	116.8	0.6%	22.7%	24.0%	484	30	4	77
CA	95 (73%)	327.2	3.1	0.7	0.1	3.1	164	2	0	0	2	49983	1307.8	2429.2	0.3%	12.5%	6.7%	1861	1	0	16
CO	13 (59%)	55.5	4.3	2.2	0.4	2.7	28	2	1	0	1	5038	146.6	159.8	0.6%	18.9%	17.4%	129	2	2	7
CT	8 (35%)	30.2	3.8	2.0	1.0	7.5	15	2	1	1	4	3831	66.9	96.0	0.4%	22.6%	15.7%	72	6	2	12
DC	3 (75%)	17.5	5.8	5.3	1.1	11.2	9	3	3	1	6	2189	44.0	40.8	0.4%	19.9%	21.5%	4	1	1	2
DE	2 (67%)	8.6	4.3	4.3	2.0	6.7	4	2	2	1	3	542	18.2	7.6	0.8%	23.7%	56.9%	19	10	6	13
FL	29 (44%)	150.1	4.8	1.9	0.2	7.2	75	2	1	0	4	20249	1928.4	739.9	0.4%	3.9%	10.1%	564	1	0	26
GA	29 (60%)	179.2	7.2	1.6	0.6	7.6	90	4	1	0	4	9565	904.9	389.1	0.9%	9.9%	23.0%	218	0	0	10
HI	3 (50%)	8.3	2.8	1.9	1.3	5.1	4	1	1	1	3	1330	20.7	26.1	0.3%	20.0%	15.9%	13	5	0	8
IA	10 (56%)	42.0	4.2	1.3	0.9	2.7	21	2	1	0	1	3426	93.5	100.0	0.6%	22.5%	21.0%	125	6	0	12
ID	4 (100%)	26.7	6.7	2.7	1.0	12.4	13	3	1	0	6	2194	89.9	32.4	0.6%	14.9%	41.3%	92	20	7	39
IL	36 (47%)	162.0	4.2	1.9	0.9	5.5	81	2	1	0	3	14690	905.7	387.8	0.6%	8.9%	20.9%	1326	19	5	40
IN	19 (44%)	89.5	4.7	0.9	0.5	3.7	45	2	0	0	2	8265	334.1	269.3	0.5%	13.4%	16.6%	237	5	0	21
KS	4 (29%)	65.8	16.4	8.2	1.1	31.8	33	8	4	1	16	2669	115.8	60.7	1.2%	28.4%	54.2%	134	22	7	61
KY	17 (89%)	97.9	5.4	4.2	1.1	7.4	49	3	2	1	4	7052	148.3	189.6	0.7%	33.0%	25.8%	484	6	2	20
LA	23 (77%)	80.9	3.5	1.6	0.5	4.5	40	2	1	0	2	6169	185.0	333.1	0.7%	21.9%	12.1%	402	7	2	23
MA	19 (44%)	99.0	5.2	3.8	1.3	8.3	50	3	2	1	4	9631	361.1	226.8	0.5%	13.7%	21.8%	622	13	0	74
ME	3 (43%)	18.8	6.2	4.6	1.5	12.7	9	3	2	1	6	2066	63.2	31.6	0.5%	14.8%	29.7%	90	24	23	43
MI	28 (52%)	191.6	6.6	3.3	1.7	8.7	96	3	2	1	4	14909	257.3	320.9	0.6%	37.2%	29.9%	1114	20	8	44
MN	17 (55%)	81.8	4.8	2.0	1.1	5.6	41	2	1	1	3	8223	114.4	138.3	0.5%	35.7%	29.6%	231	3	0	11
MO	13 (43%)	101.0	7.8	10.9	1.5	11.5	51	4	5	1	6	7631	412.0	167.2	0.7%	12.3%	30.2%	368	33	6	43
MS	10 (77%)	41.7	4.2	3.4	0.2	7.7	21	2	2	0	4	2520	209.7	201.9	0.8%	9.9%	10.3%	114	6	0	18
MT	1 (100%)	5.0	5.0	5.0	5.0	5.0	3	3	3	3	3	428	9.8	3.7	0.6%	25.6%	67.4%	22	22	22	22
NC	31 (70%)	223.9	7.0	2.3	0.5	11.5	112	3	1	0	6	17658	1134.4	457.2	0.6%	9.9%	24.5%	315	2	0	11
NE	2 (25%)	18.7	9.3	9.3	0.3	18.3	9	5	5	0	9	1101	48.2	35.5	0.8%	19.4%	26.3%	88	44	0	88
NJ	20 (40%)	64.0	3.2	1.3	0.3	3.2	32	2	1	0	2	7804	546.0	216.0	0.4%	5.9%	14.8%	269	3	0	31
NM	4 (100%)	22.8	5.7	5.7	0.8	10.6	11	3	3	0	5	2422	59.3	34.8	0.5%	19.2%	32.7%	233	58	10	107
NV	1 (33%)	0.1	0.0	0.1	0.1	0.1	0	0	0	0	0	591	55.4	52.7	0.0%	0.1%	0.1%	95	95	95	95
NY	58 (56%)	217.8	3.6	0.9	0.1	2.5	109	2	0	0	1	38567	1883.4	1145.1	0.3%	5.8%	9.5%	1406	12	2	41
OH	32 (37%)	93.8	2.9	1.1	0.2	2.7	47	1	1	0	1	12380	340.6	217.2	0.4%	13.8%	21.6%	329	4	0	7
OK	7 (54%)	50.3	7.2	8.1	0.5	11.2	25	4	4	0	6	2631	159.9	79.7	1.0%	15.7%	31.5%	251	37	18	56
OR	14 (78%)	45.9	3.3	1.0	0.2	2.4	23	2	0	0	1	5738	101.9	110.1	0.4%	22.5%	20.9%	158	3	0	12
PA	22 (29%)	170.5	7.7	2.2	1.2	9.8	85	4	1	1	5	11713	197.6	194.5	0.7%	43.2%	43.8%	462	2	0	26
RI	4 (50%)	27.3	6.8	4.5	0.8	12.9	14	3	2	0	6	2019	49.2	87.5	0.7%	27.7%	15.6%	105	29	15	38
SC	8 (42%)	75.2	9.4	6.8	0.1	15.7	38	5	3	0	8	5147	380.2	208.4	0.7%	9.9%	18.1%	175	7	2	32
SD	2 (67%)	35.7	17.9	17.9	14.6	21.2	18	9	9	7	11	1828	37.0	17.2	1.0%	48.2%	103.9%	102	51	41	61
TN	11 (55%)	137.2	11.4	6.3	0.4	21.4	69	6	3	0	11	4449	404.6	171.6	1.5%	16.9%	40.0%	517	4	0	107
TX	41 (48%)	78.2	1.9	0.6	0.2	2.1	39	1	0	0	1	15036	2974.5	1233.2	0.3%	1.3%	3.2%	838	9	1	27
UT	4 (40%)	34.3	8.6	1.9	1.2	16.0	17	4	1	1	8	2724	139.4	46.4	0.6%	12.3%	37.0%	19	3	1	9
VA	9 (26%)	117.7	13.1	12.8	4.8	15.8	59	7	6	2	8	7608	714.4	248.0	0.8%	8.2%	23.7%	32	1	1	4
VT	1 (100%)	16.1	16.1	16.1	16.1	16.1	8	8	8	8	8	1092	12.5	9.0	0.7%	64.4%	89.8%	45	45	45	45
WA	22 (71%)	106.3	4.8	2.0	0.7	8.3	53	2	1	0	4	10666	227.3	294.2	0.5%	23.4%	18.1%	248	2	0	18
WI	14 (30%)	111.2	7.9	5.3	1.4	7.0	56	4	3	1	3	7283	227.3	98.7	0.8%	24.5%	56.3%	633	35	14	54
WV	5 (63%)	17.7	3.5	2.4	1.9	4.9	9	2	1	1	2	2079	46.2	44.4	0.4%	19.2%	19.9%	151	38	15	41

© 2019 Conti RM et al. *JAMA Network Open.*

Source: Authors calculations based on data constructed and described in eTable 1. The sample includes non-profit and public general acute care hospitals participating in 340B in each year. State is a variable reported in the 340B Provider list. Contract pharmacies were identified and counted using the 340B provider list which reports the existence and name of each active contract pharmacy relationships the 340B participating hospital has in each year. Note: Revenue is defined as the amount hospitals receive for outpatient physician-administered drug treatment covered under Part B and paid by Medicare and Medicare beneficiaries. Profits are starred to remind readers that the figures presented are estimated based on an assumption that the cost of drugs under 340B are equal to 50 percent of revenue. Net operating revenue is revenue from patient operations net of contractual allowances, which are negotiated discounts from insurers. The operating margin is net operating revenue, less expenses, divided by net operating revenue, and it represents the share of net operating revenue that can be retained as profits.

© 2019 Conti RM et al. *JAMA Network Open.*
